# Alert to Action: Implementing Artificial Intelligence–Driven Clinical Decision Support Tools for Sepsis

**DOI:** 10.31486/toj.22.0098

**Published:** 2023

**Authors:** Alexander Fixler, Blake Oliaro, Marshall Frieden, Christopher Girardo, Fiona A. Winterbottom, Lisa B. Fort, Jason Hill

**Affiliations:** ^1^ The University of Queensland Medical School, Ochsner Clinical School, New Orleans, LA; ^2^ Department of Family Medicine, LCMC Health, New Orleans, LA; ^3^ Louisiana State University Health Sciences Center–Shreveport, Shreveport, LA; ^4^ Department of Pathology, Ochsner Clinic Foundation, New Orleans, LA; ^5^ Department of Critical Care, Ochsner Clinic Foundation, New Orleans, LA; ^6^ Department of Emergency Medicine, Ochsner Clinic Foundation, New Orleans, LA; ^7^ Department of Hospital Medicine, Ochsner Clinic Foundation, New Orleans, LA

**Keywords:** *Artificial intelligence*, *decision support system–clinical*, *sepsis*

## Abstract

**Background:** Sepsis is the leading cause of mortality among hospitalized patients in our health care system and has been the target of major international initiatives such as the Surviving Sepsis Campaign championed by the Society of Critical Care Medicine and Get Ahead of Sepsis led by the Centers for Disease Control and Prevention.

**Methods:** Our institution has strived to improve outcomes for patients by implementing a novel suite of integrated clinical decision support tools driven by a predictive learning algorithm in the electronic health record. The tools focus on sepsis multidisciplinary care using industry-standard heuristics of interface design to enhance usability and interaction.

**Results:** Our novel clinical decision support tools demonstrated a higher level of interaction with a higher alert-to-action ratio compared to the average of all best practice alerts used at Ochsner Health (16.46% vs 8.4% to 12.1%).

**Conclusion:** By using intuitive design strategies that encouraged users to complete best practice alerts and team-wide visualization of clinical decisions via a checklist, our clinical decision support tools for the detection and management of sepsis represent an improvement over legacy tools, and the results of this pilot may have implications beyond sepsis alerting.

## INTRODUCTION

Sepsis presents a significant burden to health care systems and continues to challenge the medical community despite aggressive research, investment, and innovation. The Centers for Disease Control and Prevention (CDC) estimated in 2016 that 270,000 Americans die each year because of sepsis.^1^ According to a study published in *Critical Care Medicine*, sepsis costs from the admission of Medicare patients to all US acute care hospitals and to skilled nursing facilities, excluding costs associated with the Veterans Administration, increased from $27.7 billion to $41.5 billion per annum from 2012 to 2018.^2^ Sepsis is currently defined under the most recent (2016) Society of Critical Care Medicine Sepsis-3 guidelines as “life-threatening organ dysfunction due to a dysregulated host response to infection.”^3^ Sepsis remains difficult to predict and manage because of the pathophysiologic processes of developing sepsis and the time-dependent nature of its treatment trajectory.

Sepsis has been the target of many international initiatives including the Society of Critical Care Medicine Surviving Sepsis and the CDC Get Ahead of Sepsis campaigns.^1,4^ These initiatives focus on detection, screening, and timely management of patients with sepsis. Yet despite these campaigns, the rate of sepsis mortality has not declined significantly.^5^ Recent innovations in sepsis detection and timely management have used digital solutions such as clinical decision support (CDS) tools—aids that guide clinical decision-making—that are integrated into the electronic health record (EHR). EHR-integrated CDS tools have been suggested as a modality to improve sepsis detection and management; however, past initiatives have shown mixed results.^6,7^ The ability of predictive algorithms to facilitate accurate and rapid assessment of risk in potentially septic patients has been well described in the literature.^8^ Pitfalls of EHR-integrated CDS tools are often related to interaction, implementation, and engagement.^9^ Examples of specific pitfalls include hospital cultures that are resistant to the adoption of new workflows, lack of visibility, and providers who are dismissive of interaction with CDS tools.

In this article, we present a novel workflow that begins with EHR-integrated predictive alerts to detect patients at risk of sepsis followed by end-user direction via intuitive CDS tools and an optimized user interface. We describe the phased development and implementation of a sepsis predictive model project including user interaction with CDS tools, response to screening, and subsequent protocolized actions.

## METHODS

The first phase in the development of the sepsis predictive model was validation in the Ochsner patient population. For the validation, we decided to exclude patients in critical care units. Epic Systems Corporation initially developed the early detection of sepsis algorithm.^10^ During the 6-month validation period, the area under the curve (AUC) for detection of sepsis was found to be noninferior in the Ochsner patient population compared to the larger Epic Systems Corporation population and was therefore used for the pilot.^10^ The Appendix provides information on the performance of the model, including the AUC in the Ochsner patient population.

The second phase in the development of the sepsis predictive model was to assign a category of risk for sepsis to patients based on recommendations and data acquired during validation. Patients were qualified by the model into 4 risk categories: low risk, medium risk, high risk, and very high risk.^10^ To reduce alert fatigue, interruptive alerts were only displayed to the care teams of patients categorized as high risk or very high risk.

During the third phase, CDS tools were further developed in-house by the Information Services team in conjunction with physicians, clinical informaticians, and nursing staff. These tools consisted of best practice alerts (BPAs) and a real-time, user-facing checklist with a timer that was integrated into the EHR and visible across care settings. Two BPAs were developed: a BPA for providers and another for nurses. BPAs fired on patients at high risk or very high risk for sepsis. The BPA color scheme was orange for high risk and red for very high risk. Clinical information, such as vital sign ranges and laboratory data, was provided in the BPA.

The provider BPA fired when orders or notes were entered and presented a simple statement, “This patient is considered at high risk or very high risk of sepsis.” The BPA prompted the provider to activate the inpatient sepsis protocol and initiate the sepsis timer. To allow for clinician decision-making autonomy, opt-out choices were available: (1) I do not believe the patient clinically has sepsis, (2) Patient is receiving adequate treatment, (3) The patient is on comfort measures, and (4) Chart review. Options 1 and 2 suppressed the BPA for 24 hours, option 3 suppressed the BPA for the rest of the encounter, and option 4 suppressed the BPA for that clinician only for 24 hours. Suppression logic was preemptively placed in the BPA workflow to reduce alert fatigue for patients who were already on antibiotics, in an intensive care unit, on inpatient hospice, or in the emergency department (ED) but not admitted to the hospital.

**Figure 1.**
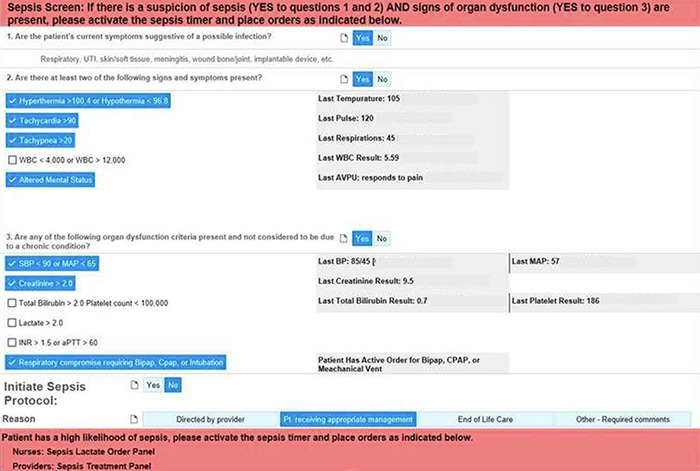
**Inpatient sepsis best practice alert for nurses.** aPTT, activated partial thrombosis clotting time; AVPU, awake, verbal, pain, unresponsive; Bipap, bilevel positive airway pressure; BP, blood pressure; CPAP, continuous positive airway pressure; INR, international normalized ratio; MAP, mean arterial pressure; Pt, patient; SBP, systolic blood pressure; UTI, urinary tract infection; WBC, white blood cell count.

The nursing BPA consisted of a set of screening questions that queried common sepsis clinical markers that encouraged users to consider the pathologic process of sepsis in decision-making and to actively initiate the nursing sepsis protocol (Figure 1). The nurse was required to answer 3 sequential, linear questions: (1) Are the patient's current symptoms suggestive of a possible infection?, (2) Are there at least two of the following signs and symptoms present?, and (3) Are any of the following organ dysfunction criteria present and not considered to be due to a chronic condition? The nursing BPA automatically displayed data from the patient's chart to highlight potential signs/symptoms of infection, including fever/hypothermia, tachycardia, tachypnea, and altered mental status. Laboratory values indicative of organ dysfunction, including renal function, coagulopathy, liver function, and abnormal blood gas, were displayed. Abnormal values were visually highlighted. If the nurse answered yes to all 3 questions, the nurse was prompted to initiate a nursing sepsis protocol. A nurse-driven sepsis protocol allowed nurses to order serum lactates, increase vital sign monitoring, and activate the sepsis timer. The nurse screening opt-out questions that did not result in the initiation of inpatient sepsis treatment protocol were (1) Directed by provider, (2) Patient receiving appropriate management, (3) End of life care, and (4) Other - required comments. If any questions were answered no, further alerts were suppressed for the next 24 hours.

**Figure 2.**
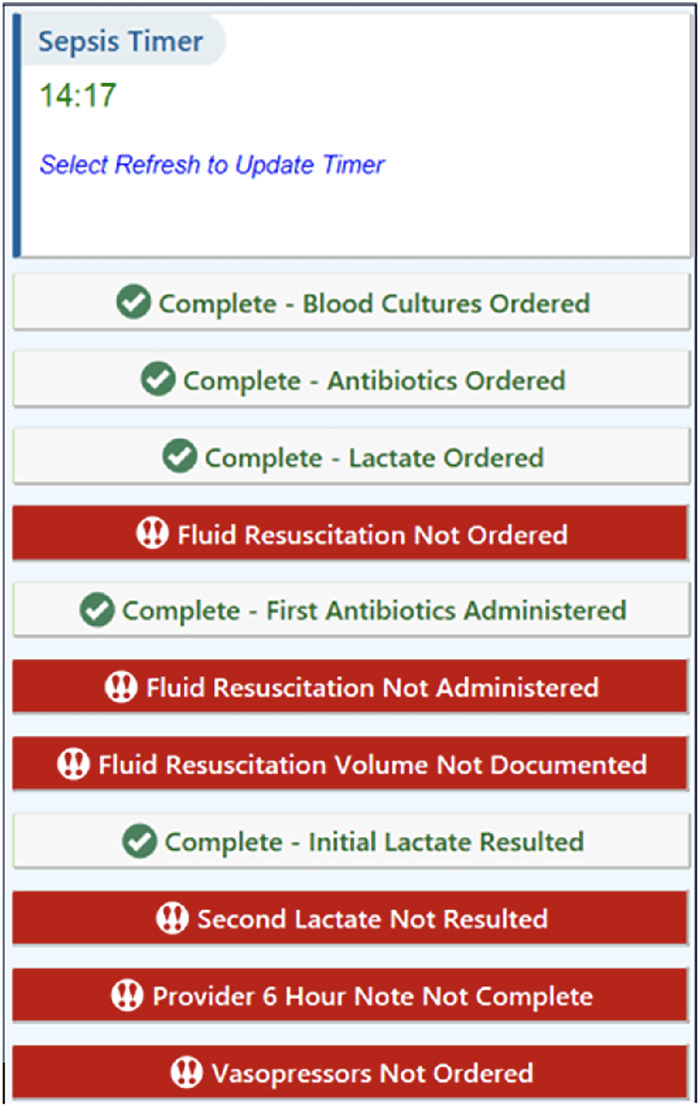
Shared sepsis checklist and timer.

The sepsis checklist and timer (Figure 2) was designed to be initiated by any care team member. The timer alerts the multidisciplinary team across care settings to the initiation of the sepsis checklist and completed actions.

The BPA and the checklist and timer were created with industry-standard heuristics of user interface design in mind. Nielsen's 10 principles were used to guide the design of the CDS tools and improve them throughout design and implementation.^11^ For example, visibility of the tools in the EHR was prioritized to include all members of the treatment team to build trust and cohesion through continuous communication of patient care. Multiple plan-do-study-act (PDSA) cycles, user surveys, and user tests resulted in improved wording and graphic design of the interface over time, and work is still ongoing to determine the best alert-to-action ratios. Overall, the strategy of drawing clinician interest by BPA visualization of relevant/abnormal data was paramount, as well as limiting the false-positive firing of BPAs based on the suppression criteria outlined above.

**Figure 3.**
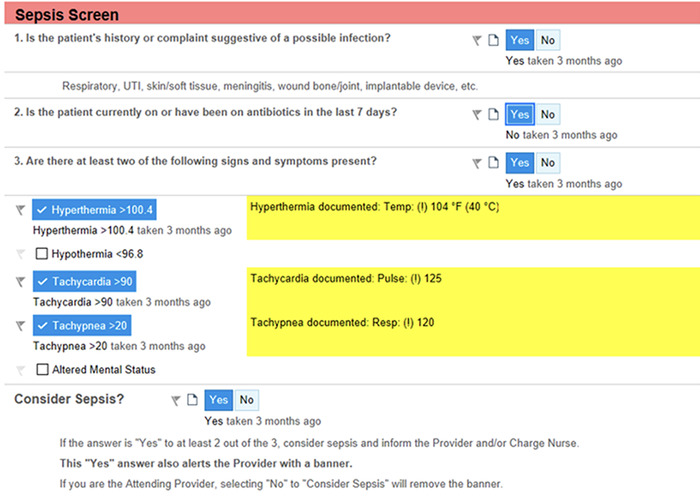
**Emergency department sepsis screening questionnaire.** Resp, respiration; Temp, temperature; UTI, urinary tract infection.

The sepsis predictive algorithm was not used in the ED, as limited data at triage precluded accurate risk assessment by the algorithm. Instead, a screening questionnaire (Figure 3) was provided at triage to the intake nurse if the patient had abnormal vital signs (fever/hypothermia, tachycardia, tachypnea, or altered mental status). If questions were answered in the affirmative, a sepsis banner was activated to draw the attention of the ED provider and nurse and prompt the provider to order a sepsis panel. If the sepsis panel was ordered, the ED protocol was activated, with the sepsis checklist and timer displayed as a sidebar on the right-hand pane of the EHR (Figure 2). The usage of the sepsis checklist and timer will be detailed in subsequent studies looking at the timing of specific actions once the patient is identified as septic, but that analysis was outside the scope of this initial study.

Phase 4 involved implementation of the predictive model and CDS tools in a stepwise approach across the hospital system from June 29, 2021, through November 15, 2022. PDSA cycles occurred weekly during the pilot as tools were optimized using health care provider feedback during implementation. Enhancement focused on usability and user interaction with the complex suite of tools, screening volume, and sepsis protocol initiation. Iterative PDSA cycles included implementation at 3 EDs in October 2021, followed by inpatient settings at the same hospitals in November 2021 and January 2022. The study dates for this project were the 6-month period from March 1, 2022, through September 1, 2022, due to most current data collection and interpretation. Data were collected from the 14 Ochsner facilities where the new predictive model and CDS tools were deployed (Table 1).

**Table 1.** Implementation Timeline of Clinical Decision Support Tools

Location	Emergency Department Implementation Date	Inpatient Implementation Date
Ochsner Medical Center–Kenner, Kenner, LA^a^	June 29, 2021	June 29, 2021
Ochsner Baptist Hospital, New Orleans, LA^a^	October 5, 2021	November 16, 2021
Ochsner Medical Center–West Bank, Gretna, LA^a^	October 12, 2021	November 18, 2021
Ochsner Medical Center–North Shore, Slidell, LA^a^	October 26, 2021	January 24, 2022
St. Bernard Parish Hospital, Chalmette, LA^a^	December 7, 2021	January 11, 2022
Ochsner Medical Center, New Orleans, LA^a^	January 11, 2022	March 29, 2022
Ochsner Medical Center–Baton Rouge, Baton Rouge, LA^a^	February 15, 2022	March 15, 2022
St. Charles Parish Hospital, Luling, LA^a^	March 15, 2022	March 15, 2022
Leonard J. Chabert Medical Center, Houma, LA^a^	March 15, 2022	March 15, 2022
Ochsner St. Anne Hospital, Raceland, LA^a^	March 15, 2022	March 15, 2022
Ochsner Medical Center–Hancock, Bay St. Louis, MS^a^	March 15, 2022	March 15, 2022
Ochsner Lafayette General Medical Center, Lafayette, LA^a^	May 1, 2022	May 1, 2022
Ochsner LSU Health Shreveport–Academic Medical Center, Shreveport, LA and Ochsner LSU Health–Monroe Medical Center, Monroe, LA^a^^,^^b^	July 19, 2022	July 19, 2022
Slidell Memorial Hospital, Slidell, LA^a^	August 9, 2022	August 9, 2022
Ochsner Rush Health, Meridian, MS and Ochsner LSU Health–St. Mary Medical Center, Shreveport, LA	September 13, 2022	September 13, 2022
St. Tammany Parish Hospital, Covington, LA	October 17, 2022	October 17, 2022
Terrebonne General Medical Center, Houma, LA and Titus Regional Medical Center, Mount Pleasant, TX	November 15, 2022	November 15, 2022

^a^Facilities included in the study period analysis.

^b^Data from Ochsner LSU Health Shreveport–Academic Medical Center and Ochsner LSU Health–Monroe Medical Center were pooled and analyzed as a single site.

LSU, Louisiana State University.

Outcomes of the project were quantified in a variety of ways. The first was analysis of the alert-to-action ratio—the ratio of end users receiving system-generated alerts about their patients via BPA to any action logged subsequent to the alert—on triggered BPAs. The output of the BPA screening was recorded as 1 of 3 independent endpoints: (1) screenings not completed, (2) inpatient treatment protocol not initiated, and (3) inpatient treatment protocol initiated. For providers, the inpatient treatment protocol was a sepsis panel order, and for nurses, it was a lactate panel order. Process metrics were compared by time and action percentage on patients who were considered to be septic based on inclusion criteria determined by initiation of the sepsis inpatient protocol. These parameters were then compared to a retrospective review of the same set of patients identified by a structured query language (SQL) dataset to look at model performance and subsequent patient inclusion for various process steps. The SQL data were externally validated against a well-known national quality database (Vizient Inc) to ensure accuracy of patient identification and process metrics, including time zero. Outcome metrics measured were primary and secondary sepsis risk-adjusted mortality index (RAMI) and were reported by standard inpatient quality outcome data. Metrics were also compared to historic cohorts and externally validated against similar facilities across the country through Vizient.

Ochsner Institutional Review Board approval was granted to our project. Data for our project were deidentified prior to upload into Tableau-connected (Tableau Software LLC) databases. No agents of this study had financial obligations that could result in bias.

## RESULTS

Data were collected and aggregated across the 14 active Ochsner Health facilities from March 1, 2022, to September 1, 2022. Active facilities were sites where the CDS tools were available as outlined in Table 1. Because the sepsis BPAs were only active inside the noncritical care hospital environment, intensive care units and EDs were excluded. Proactive screenings not done from the BPA workflow were also excluded. The alert-to-action metrics, including frequency and type of alerting, were matched to patient risk categories and clinical departments.

**Figure 4.**
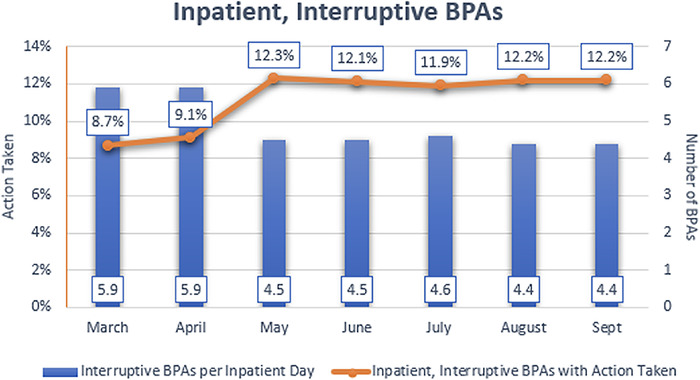
Average number of best practice alerts (BPAs) fired per day compared to the percentage of BPAs with action taken per day in the inpatient setting. The numbers in boxes above each month represent the average number of BPAs that were fired per day in that month.

Figure 4 shows the mean alert-to-action ratios for BPAs across the 14 hospitals in the Ochsner Health system where the model and CDS tools had been deployed. Overall BPAs per inpatient day decreased, while alert-to-action ratios increased during the study time frame, from an average of 8.7% in March to 12.2% in September. This alert-to-action ratio represents the top decile of all Epic users and is considered a best practice in CDS.

Interaction with the sepsis screening form was quantified by the completion of the 3 yes/no screening questions shown in Figure 1. The results of BPA screening interaction data are summarized in Table 2. From March 1, 2022, to September 1, 2022, a total of 1,959 sepsis screenings were initiated, and 237 patients were screened as triple-yes, meaning the screener answered yes to all 3 questions, confirming a clinical suspicion of sepsis. Of those, 39 patients (16.46%) had the sepsis treatment protocol initiated. Inpatient treatment was not initiated on 192 patients, the majority of whom (94.79%) were already receiving appropriate management. In 3 cases, users completed the screening questions but did not submit the answers; these cases were captured as null responses and, for the purpose of this study, were categorized as negative screenings. Sepsis screening was not completed for 6 patients (2.53%).

**Table 2.** Triple-Yes Patient Sepsis Screening Interaction Results, March 1, 2022, to September 1, 2022

Screen Result		Screen Completed							
Treatment Protocol Result	Screen Not Completed	Treatment Protocol Not Initiated						Treatment Protocol Initiated	
Reason Treatment Protocol Not Initiated	Total	Patient Receiving Appropriate Management	End of Life Care	Directed by Provider	Other	Null	Total	Total	Grand Total
**Percentage of Screens**	2.53%	76.79%	1.27%	0.84%	0.84%	1.27%	81.01%	16.46%	100%
**Discrete Screens, n**	6	182	3	2	2	3	192	39	237

**Figure 5.**
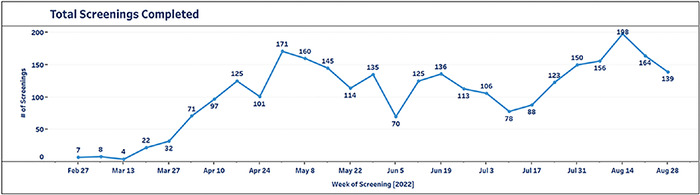
Total sepsis screenings completed per week during the study period.

Weekly screening completion values are displayed in Figure 5. The number of screenings completed weekly during the observation period increased from 7 the week of February 28 to 139 the week of August 28, with a maximum number of screenings occurring the week of August 14 at 198. An increasing completion trend is further observed in the average number of screenings per month. For the month of March, the mean number of screenings completed was 16.5 compared to 164.25 in August, attributable in part to the rollout of the CDS sepsis toolkit at additional hospital facilities, including the largest academic medical centers, Ochsner Medical Center in New Orleans, Louisiana, and Ochsner LSU Health Shreveport–Academic Medical Center in Shreveport, Louisiana, in January, March, and July.

## DISCUSSION

Ochsner Health is a large health care system with unique clinical environments that span the spectrum from small community hospitals to large quaternary academic centers. Novel CDS tools in conjunction with the predictive algorithm for sepsis detection can compensate for the fewer resources and lower acuity presentations in smaller hospitals. In such settings, conducting labor-intensive bedside screenings of all admitted patients for sepsis is not often a priority, as the cost-benefit ratio is small. With this new set of tools, all patients can be screened without the need to invest in additional staff or intensive training, helping to keep costs low while maintaining high-quality care. Given that Ochsner Health is a rapidly expanding health system, these tools are flexible enough to be implemented at any location and are intuitive so that intensive training is not required. The stepwise, linear nature of the screening tool in conjunction with the checklist and timer equips both nurses and physicians with crucial information regarding sepsis detection, decision-making, and management.

Convergent workflows in the EHR between physicians and nurses in which a positive screening from nursing prompts the physician and vice versa promote accountability within the care teams. By making the time zero from a positive screening explicit and visible to the clinical care team in both the nursing and provider workflows and across care settings, the hypothesis is that this visibility will not only promote shared responsibility for patient care but also encourage rapid treatment for all process endpoints and subsequent improvement of primary sepsis RAMI. Our research team and the Ochsner Health informatics staff emphasis on user-centered design was paramount in driving real-world human behavior modification based on the output of a predictive algorithm. These solutions helped to improve our noise-to-signal ratio, while alleviating some of the issues surrounding alert fatigue that further increased compliance with sepsis standards of care.

This set of CDS tools demonstrated higher levels of interaction than other system BPAs, as 16.46% of patients for whom a sepsis BPA was initiated received an action subsequent to the BPA compared to an average alert-to-action ratio of 8.4% to 12.1% for all BPAs. Only a small percentage of end users for whom the sepsis screening BPA fired did not complete the screening. Utilization of the CDS tools increased from the beginning of the observation period to the end. We hypothesize that this increase could be attributed to cultural buy-in, increasing familiarity with the tools, and subsequent improvement in the BPA usability through multiple PDSA cycles.

Implementations of sepsis detection and management tools created using the Epic Systems Corporation proprietary Cognitive Computing Model: Early Detection of Sepsis model have fallen short of expectations.^10,12^ While the validity of the aforementioned sepsis predictive algorithm came into question in 2021, implementation of the model into existing workflows is fundamental to its success.^13^ By creating intuitive, integrated, and collaborative CDS tools, we improved intra-institutional interaction, based on end-user feedback and heuristic testing, over previous attempts at CDS tools implementation. Successful implementation may therefore be considered independent of model characteristics or model use case, and this project provides a distinct framework for future model iteration implementations without necessitating ground-up project builds.

Improved utilization and interaction with the CDS tools also has downstream effects on future iterations of the tools themselves. Previous electronic support alert attempts at Ochsner Health had little integration with the EHR, so actionable data on provider interaction with the tools could not be extracted or observed. The sepsis CDS tools allow for objective analysis of the clinician's thought process through data inputs in near-real time. Previously, this kind of clinical action data could only be studied retrospectively by abstracting data from medical records. Such manual data collection is time-consuming and labor intensive. The new tools supply researchers with data on how clinicians perceive and manage sepsis. This information can inform further innovation, as the CDS tools can be modified to maximize efficiency and management compliance based on robust input data. Dashboards in the organization's data visualization solution provided rapid feedback, facilitating adjustments and tracking utilization across the health system. These dashboards were used from the earliest stages of the CDS tool implementation, as they provided valuable information on interruptive alerts and called attention to signs of alert fatigue and low engagement.

Implementation of the sepsis predictive algorithm, CDS, and BPAs was done in conjunction with additional workflows, including virtual nurse screening of a sepsis rounding list. This list displays data populated by the predictive algorithm and is monitored by a team of nurses responsible for identifying and proactively screening patients at high risk of sepsis. This nurse user group did not directly receive BPAs and was therefore excluded from the analysis. This dual implementation strategy was intended to ensure levels of redundancy in sepsis screening and improve identification of sepsis in patients. This redundancy meant, however, that the majority of institutional screenings were completed by the dedicated virtual team and not by the end users evaluated in this study. The virtual nurse screenings could have had an impact on the interaction results, as a large number of screenings that would have otherwise been made available to bedside end users had already been completed by the virtual nurse rounding team.

As the tools were implemented, we observed a population of end users who completed the BPA screening, answered yes to components of the screening that require action, but took no subsequent action. In Table 2, these cases are reported as triple-yes with null values. In addition, some end users appear to have found a way to exit the screening questions without completing the screen. Whether this action was intentional is unclear, but this action was observed in 2.53% of total screenings undertaken and may have had an impact on the alert-to-action ratio, as this population of users was alerted, interacted with the tools, but did not initiate any action. Therefore, our alert-to-action ratio might be underestimated.

## CONCLUSION

Creating CDS tools that engage end users and promote interaction can be crucial to implementation of EHR predictive models in any health system and in numerous contexts. By using intuitive design strategies that encouraged users to complete BPAs and team-wide visualization of clinical decisions via a checklist, our CDS tools for the detection and management of sepsis represent an improvement over legacy tools in terms of interaction and engagement. The results of this pilot may have implications beyond sepsis alerting and can be applied to future implementations of CDS tools for various pathologies. We plan to conduct further research to assess the efficacy of the CDS tools with regard to compliance with management bundles and to capture additional primary outcomes, such as sepsis RAMI.

## Figures and Tables

**Figure A1.**
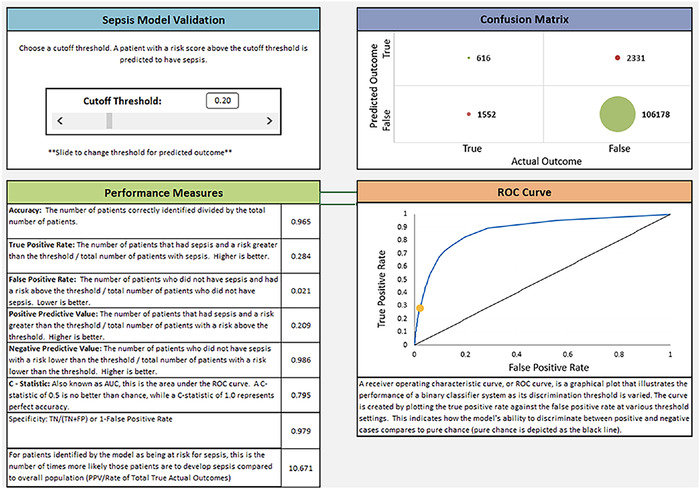
**Sepsis receiver operating characteristic (ROC) curve and performance on trained cutoff thresholds.** AUC, area under the curve; FP, false positive; PPV, positive predictive value; TN, true negative.

**Figure A2.**
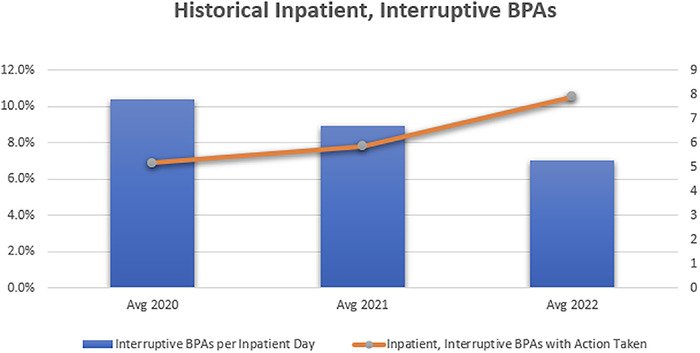
Average (Avg) number of all inpatient best practice alerts (BPAs) and action ratings, 2020 to 2022.

**Figure A3.**
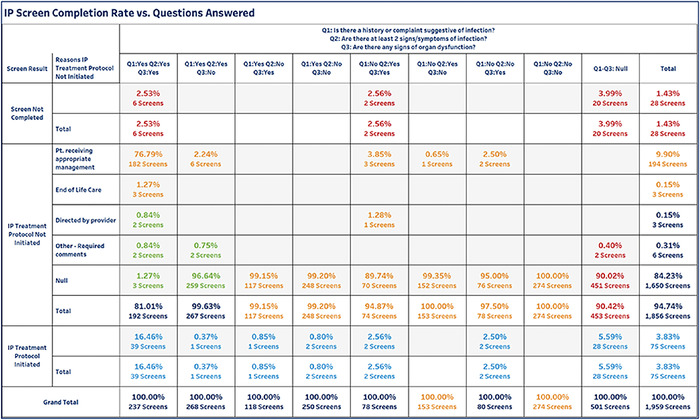
**Complete sepsis screening response data.** IP, inpatient; Pt, patient; Q, question.

## Notes

*This article meets the Accreditation Council for Graduate Medical Education and the American Board of Medical Specialties Maintenance of Certification competencies for Patient Care, Medical Knowledge, and Systems-Based Practice.*
